# Supplementary material for: Improved injury detection through harmonizing multi-site neuroimaging data after experimental TBI: a Translational Outcomes Project in Neurotrauma consortium study
Source: Front Neurol. 2025 Aug 20;16:1612598. doi: 10.3389/fneur.2025.1612598 (PMC12406496; doi:10.3389/fneur.2025.1612598)
Supplement: Supplementary file 1 [file Data_Sheet_1.DOCX]

Supplementary Figures Legend

**Fig. S1. Effect of univariate and voxel-level harmonization on the burden of whole brain injury volume using pooled sham data: AD.** Brain volumes of pathology identified by (**A,C**) AD_Low_ and (**B,D**) AD _High_ at 3d (**A,B**) and 30d (**C,D**) are plotted for each site before (“Original”) and after univariate and voxel-level harmonization. ###/*** = a significant effect of location and injury, respectively, p<0.001, ##/** = p<0.01, #/* = p<0.05 (linear mixed model ANOVA).

**Fig S2. Harmonization at voxel-level resolution: AD. (A-B**) Harmonization of AD values of each individual voxel across site pairs is demonstrated using Bland-Altman plots for (**A**)original, unharmonized values and (**B**) harmonized values. The difference between sites decreases due to harmonization.

**Fig S3. Whole-brain maps of regional changes in power due to harmonization: AD. (A)** Difference in power across a pooled average of all three sites post-injury due to harmonization and **(B)** across site and post-injury day. The magnitude scale -30% and 30% indicates the percent difference in statistical power between pre and post voxel-level harmonization, where yellow/blue indicate power increases and decreases, respectively due to harmonization.

**Fig. S4. Whole-brain maps of regional changes in effect size due to harmonization: AD. (A)** Difference in effect size across a pooled average of all three site post-injury due to harmonization and **(B)** across site and post-injury day. The magnitude scale –1 and +1 Cohen’s d indicates the difference in effect size/voxel between pre and post voxel-level harmonization, where yellow/blue indicate power increases and decreases, respectively due to harmonization.

**Fig. S5. Multi-Site Voxel overlap maps delineating regions of common pathology due to data harmonization: AD. V**oxel overlap maps derived from whole-brain data across all 3 sites showing the incidence of the number of rats at each voxel location where AD values differ significantly from pooled sham data **(A)** before (Original) and **(B)** after harmonization (Harmonized). Images show voxels where there was a lower (Pink) and higher (Blue/Red) proportion of rats in which the AD value was significantly different from pooled shams due to data harmonization (p<0.01, FDR corrected).

**Fig. S6. Effect of univariate and voxel-level harmonization on the burden of whole brain injury volume using pooled sham data: MD.** Brain volumes of pathology identified by (**A,C**) MD_Low_ and (**B,D**) MD _High_ at 3d (**A,B**) and 30d (**C,D**) are plotted for each site before (“Original”) and after univariate and voxel-level harmonization. ###/*** = a significant effect of location and injury, respectively, p<0.001, ##/** = p<0.01, #/* = p<0.05 (linear mixed model ANOVA).

**Fig S7. Harmonization at voxel-level resolution: MD. (A-B**) Harmonization of MD values of each individual voxel across site pairs is demonstrated using Bland-Altman plots for (**A**)original, unharmonized values and (**B**) harmonized values. The difference between sites decreases due to harmonization.

**Fig S8. Whole-brain maps of regional changes in power due to harmonization: MD. (A)** Difference in power across a pooled average of all three sites post-injury due to harmonization and **(B)** across site and post-injury day. The magnitude scale -30% and 30% indicates the percent difference in statistical power between pre and post voxel-level harmonization, where yellow/blue indicate power increases and decreases, respectively due to harmonization.

**Fig. S9. Whole-brain maps of regional changes in effect size due to harmonization: MD. (A)** Difference in effect size across a pooled average of all three site post-injury due to harmonization and **(B)** across site and post-injury day. The magnitude scale –1 and +1 Cohen’s d indicates the difference in effect size/voxel between pre and post voxel-level harmonization, where yellow/blue indicate power increases and decreases, respectively due to harmonization.

**Fig. S10. Multi-Site Voxel overlap maps delineating regions of common pathology due to data harmonization: MD.** Voxel overlap maps derived from whole-brain data across all 3 sites showing the incidence of the number of rats at each voxel location where MD values differ significantly from pooled sham data **(A)** before (Original) and **(B)** after harmonization (Harmonized). Images show voxels where there was a lower (Pink) and higher (Blue/Red) proportion of rats in which the MD value was significantly different from pooled shams due to data harmonization (p<0.01, FDR corrected).

**Fig. S11. Effect of univariate and voxel-level harmonization on the burden of whole brain injury volume using pooled sham data: RD.** Brain volumes of pathology identified by (**A,C**) RD_Low_ and (**B,D**) RD _High_ at 3d (**A,B**) and 30d (**C,D**) are plotted for each site before (“Original”) and after univariate and voxel-level harmonization. ###/*** = a significant effect of location and injury, respectively, p<0.001, ##/** = p<0.01, #/* = p<0.05 (linear mixed model ANOVA).

**Fig S12. Harmonization at voxel-level resolution: RD. (A-B**) Harmonization of RD values of each individual voxel across site pairs is demonstrated using Bland-Altman plots for (**A**)original, unharmonized values and (**B**) harmonized values. The difference between sites decreases due to harmonization.

**Fig S13. Whole-brain maps of regional changes in power due to harmonization: RD. (A)** Difference in power across a pooled average of all three sites post-injury due to harmonization and **(B)** across site and post-injury day. The magnitude scale -30% and 30% indicates the percent difference in statistical power between pre and post voxel-level harmonization, where yellow/blue indicate power increases and decreases, respectively due to harmonization.

**Fig. S14. Whole-brain maps of regional changes in effect size due to harmonization: RD. (A)** Difference in effect size across a pooled average of all three site post-injury due to harmonization and **(B)** across site and post-injury day. The magnitude scale –1 and +1 Cohen’s d indicates the difference in effect size/voxel between pre and post voxel-level harmonization, where yellow/blue indicate power increases and decreases, respectively due to harmonization.

**Fig. S15. Multi-Site Voxel overlap maps delineating regions of common pathology due to data harmonization: RD. V**oxel overlap maps derived from whole-brain data across all 3 sites showing the incidence of the number of rats at each voxel location where RD values differ significantly from pooled sham data **(A)** before (Original) and **(B)** after harmonization (Harmonized). Images show voxels where there was a lower (Pink) and higher (Blue/Red) proportion of rats in which the RD value was significantly different from pooled shams due to data harmonization (p<0.01, FDR corrected).

**Fig. S16. Effect of univariate harmonization on the burden of whole brain injury volume using pooled sham data at all four sites.** Brain volumes of pathology at all four sites identified by (**A,C**) FA_Low_ and (**B,D**) FA_High_ at 3d (**A,B**) and 30d (**C,D**) are plotted for each site before (“Original”) and after univariate and voxel-level harmonization. ###/*** = a significant effect of location and injury, respectively, p<0.001, ##/** = p<0.01, #/* = p<0.05 (linear mixed model ANOVA).

**Fig. S17. Whole-brain maps of regional changes in effect size due to harmonization across all four sites. (A)** Difference in effect size across a pooled average of all four site post-injury due to harmonization and **(B)** across site and post-injury day. The magnitude scale –1 and +1 Cohen’s d indicates the difference in effect size/voxel between pre and post voxel-level harmonization, where yellow/blue indicate power increases and decreases, respectively due to harmonization.

**Fig. S18. Multi-Site Voxel overlap maps delineating regions of common pathology due to data harmonization at all four sites. V**oxel overlap maps derived from whole-brain data across all four sites showing the incidence of the number of rats at each voxel location where FA values differ significantly from pooled data **(A)** before (Original) and **(B)** after harmonization (Harmonized). Images show voxels where there was a lower (Pink) and higher (Blue/Red) proportion of rats in which the FA value was significantly different from pooled shams due to data harmonization (p<0.01, FDR corrected).
