# Supplementary material for: Improved injury detection through harmonizing multi-site neuroimaging data after experimental TBI: a Translational Outcomes Project in Neurotrauma consortium study
Source: Front Neurol. 2025 Aug 20;16:1612598. doi: 10.3389/fneur.2025.1612598 (PMC12406496; doi:10.3389/fneur.2025.1612598)
Supplement: Supplementary file 2 [file Image_1.pdf]

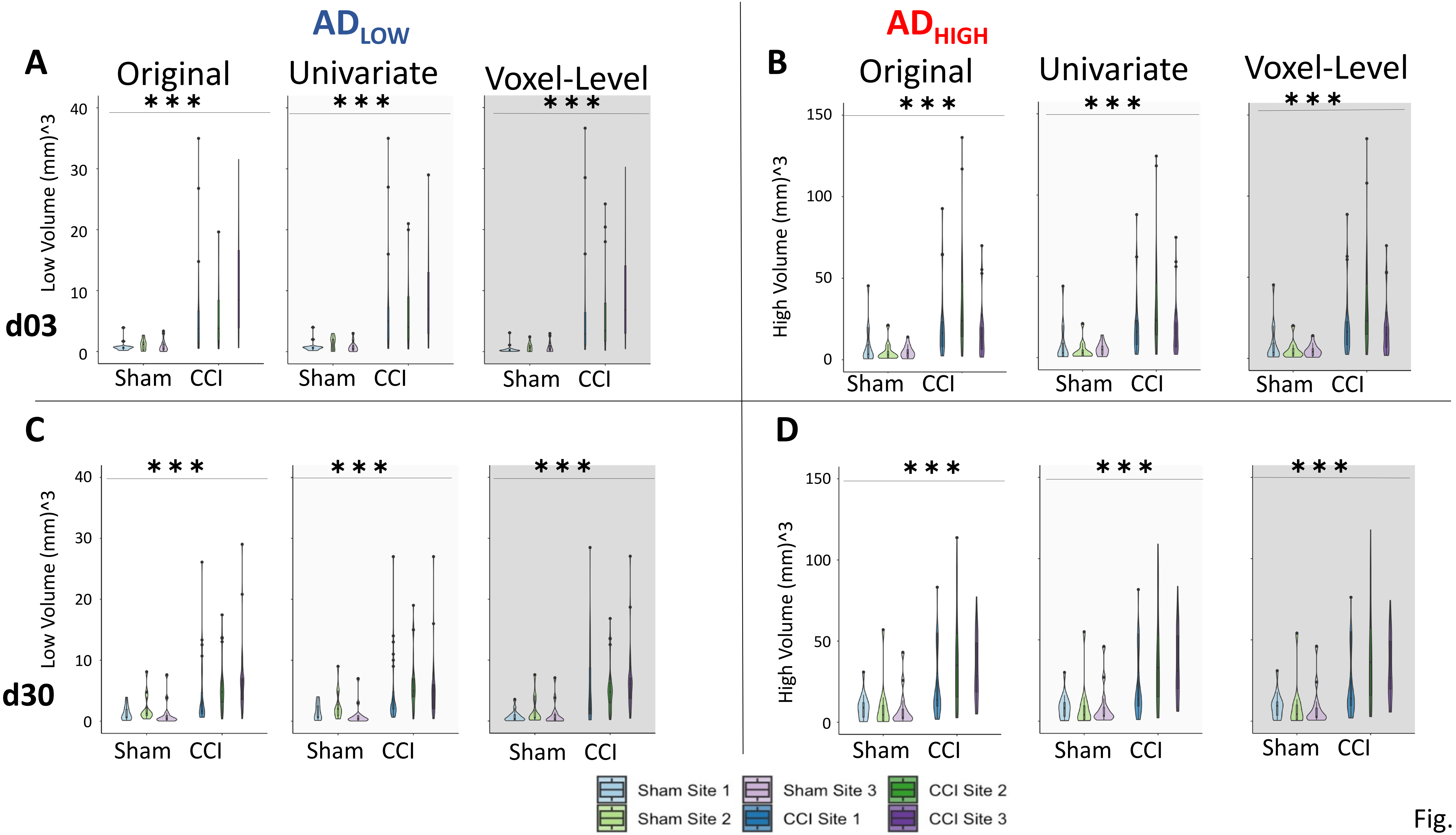

AD$_{LOW}$
AD$_{HIGH}$
A
B
C
D
Original
Univariate
Voxel-Level
d03
d30
Low Volume (mm)^3
High Volume (mm)^3
* * *
Sham
CCI
0
10
20
30
40
50
100
150
Sham Site 1
Sham Site 2
Sham Site 3
CCI Site 1
CCI Site 2
CCI Site 3

Fig. S1

Mean-Difference Plots: AD

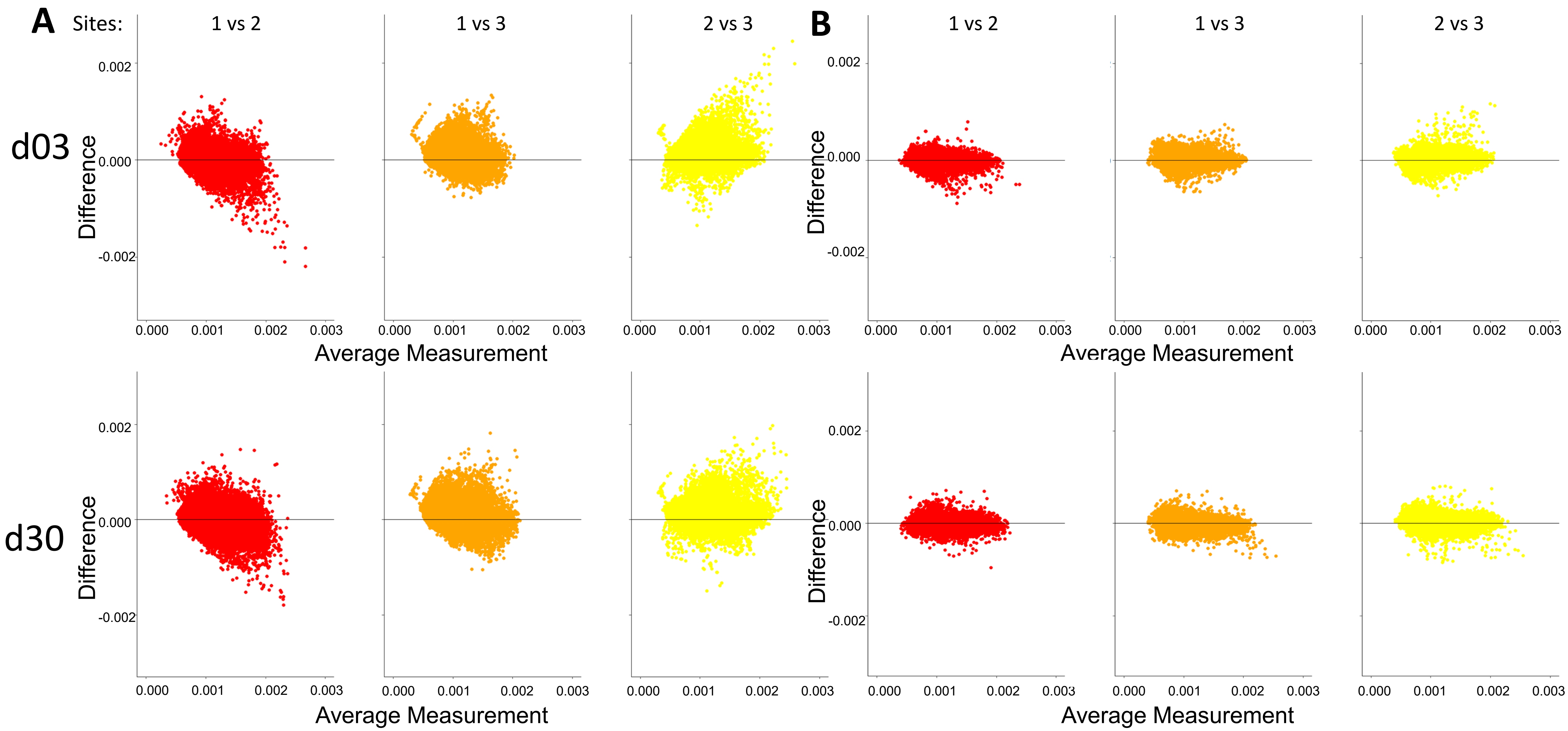

Fig. S2

Change in Power: AD

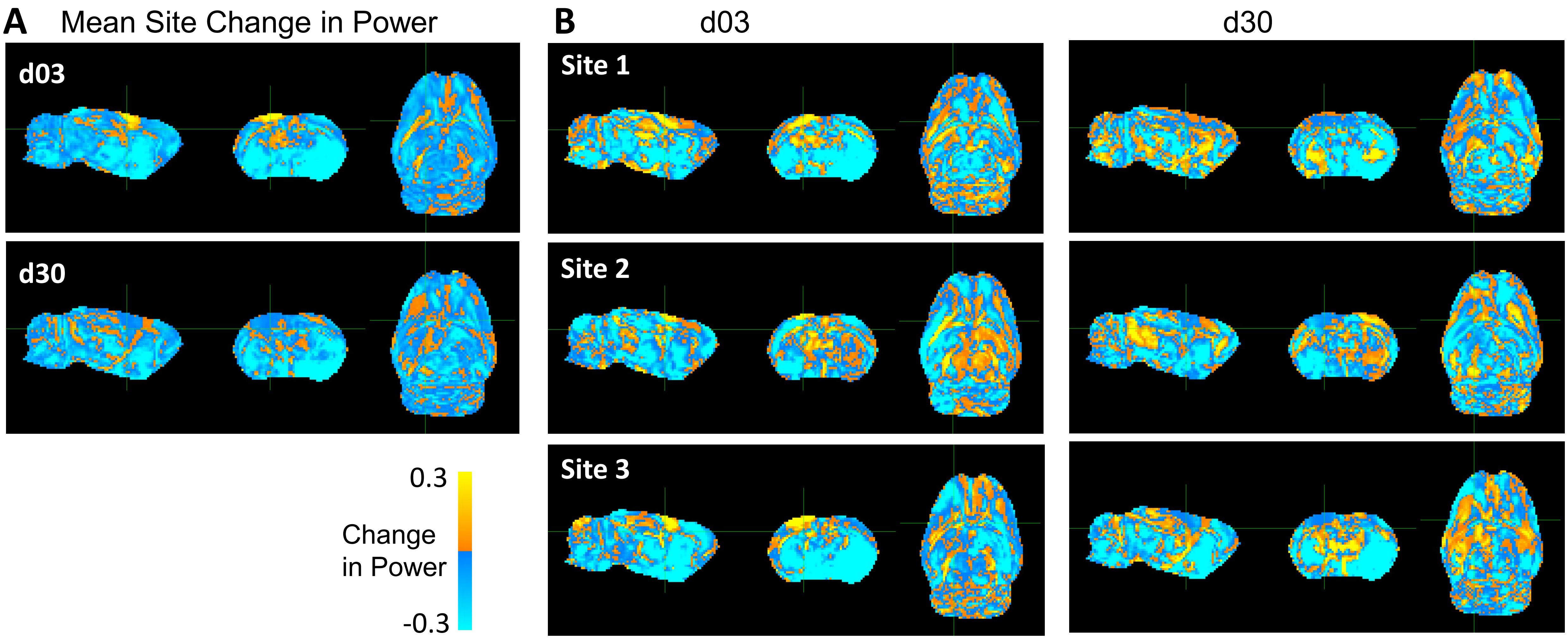

Fig. S3

Effect Size: AD

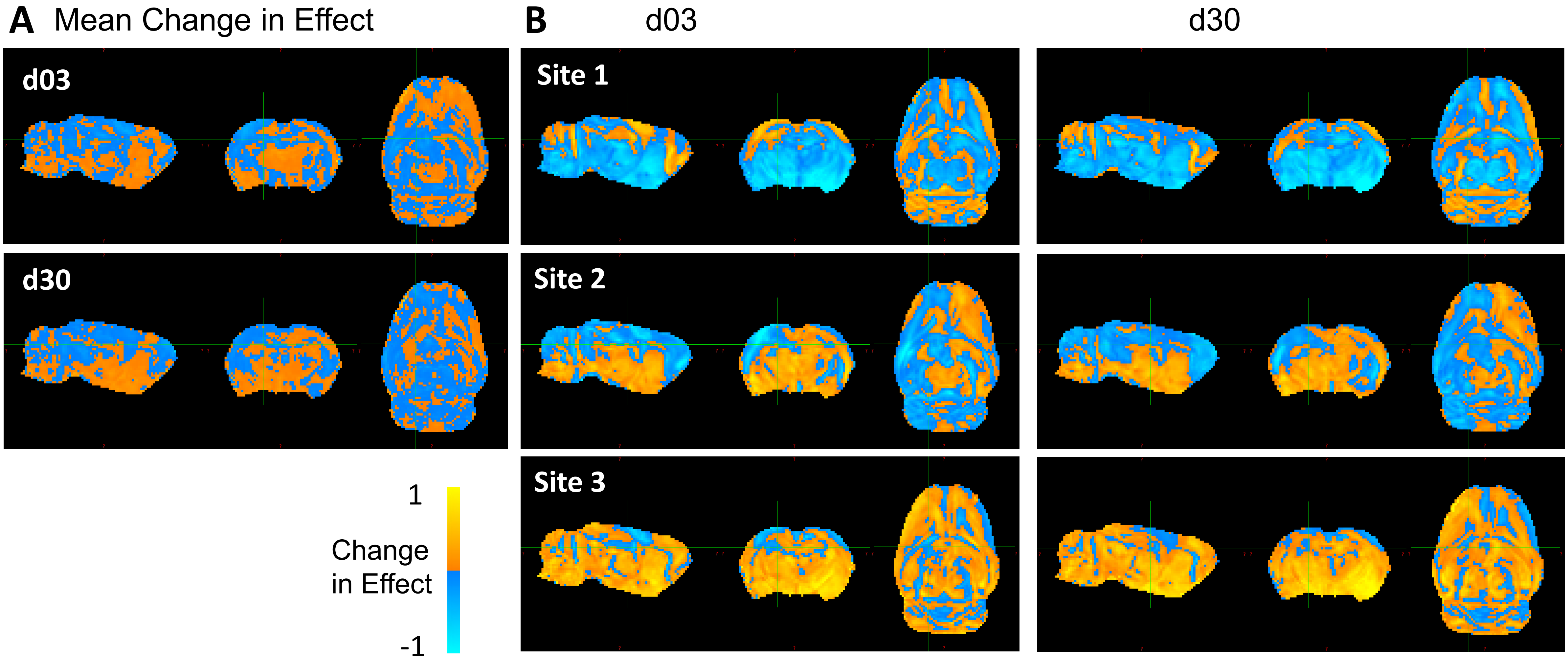

Fig. S4

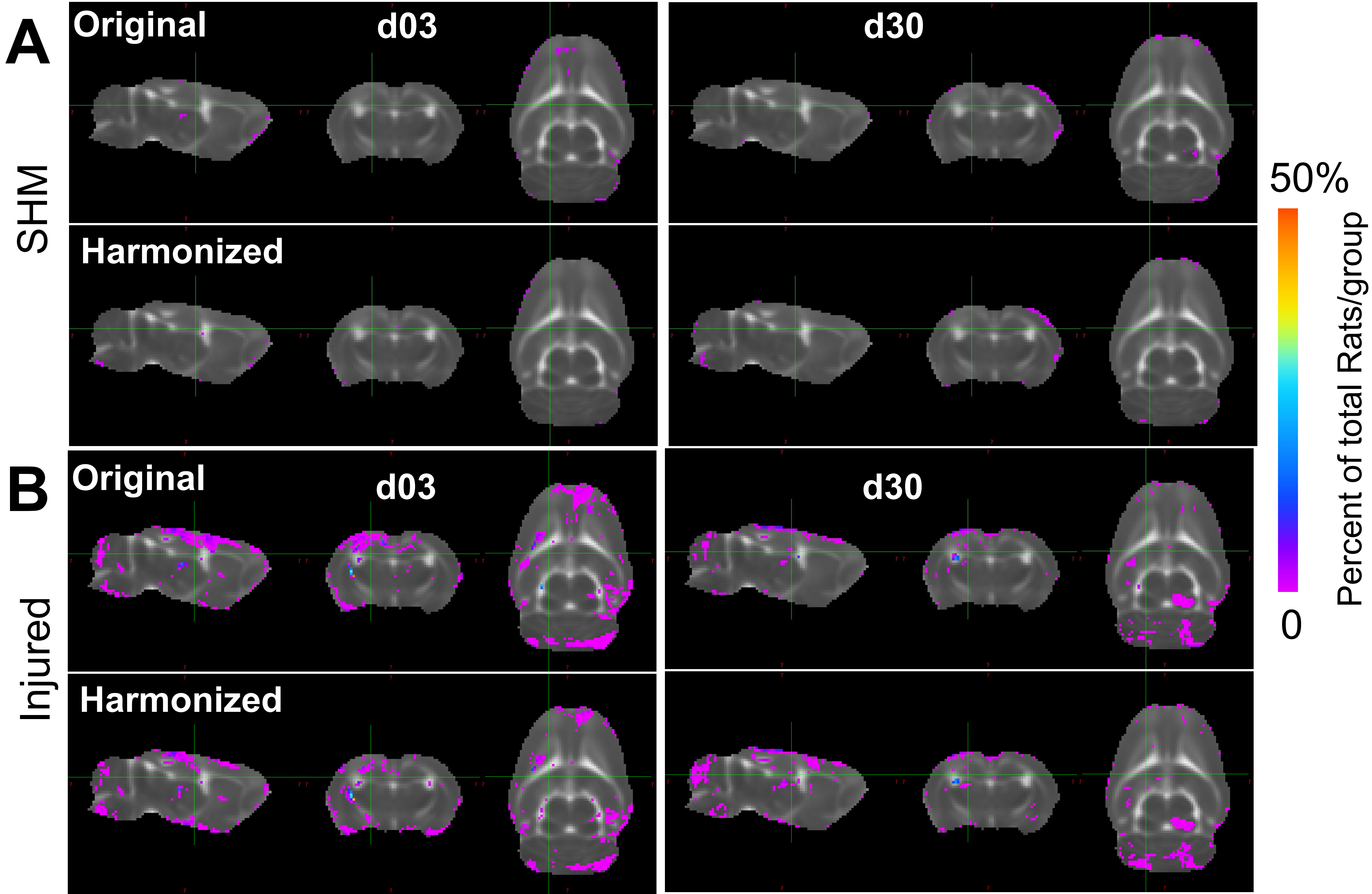
A
Original
d03
d30
SHM
Harmonized
B
Original
d03
d30
Injured
Harmonized
50%
Percent of total Rats/group
0

Fig. S5

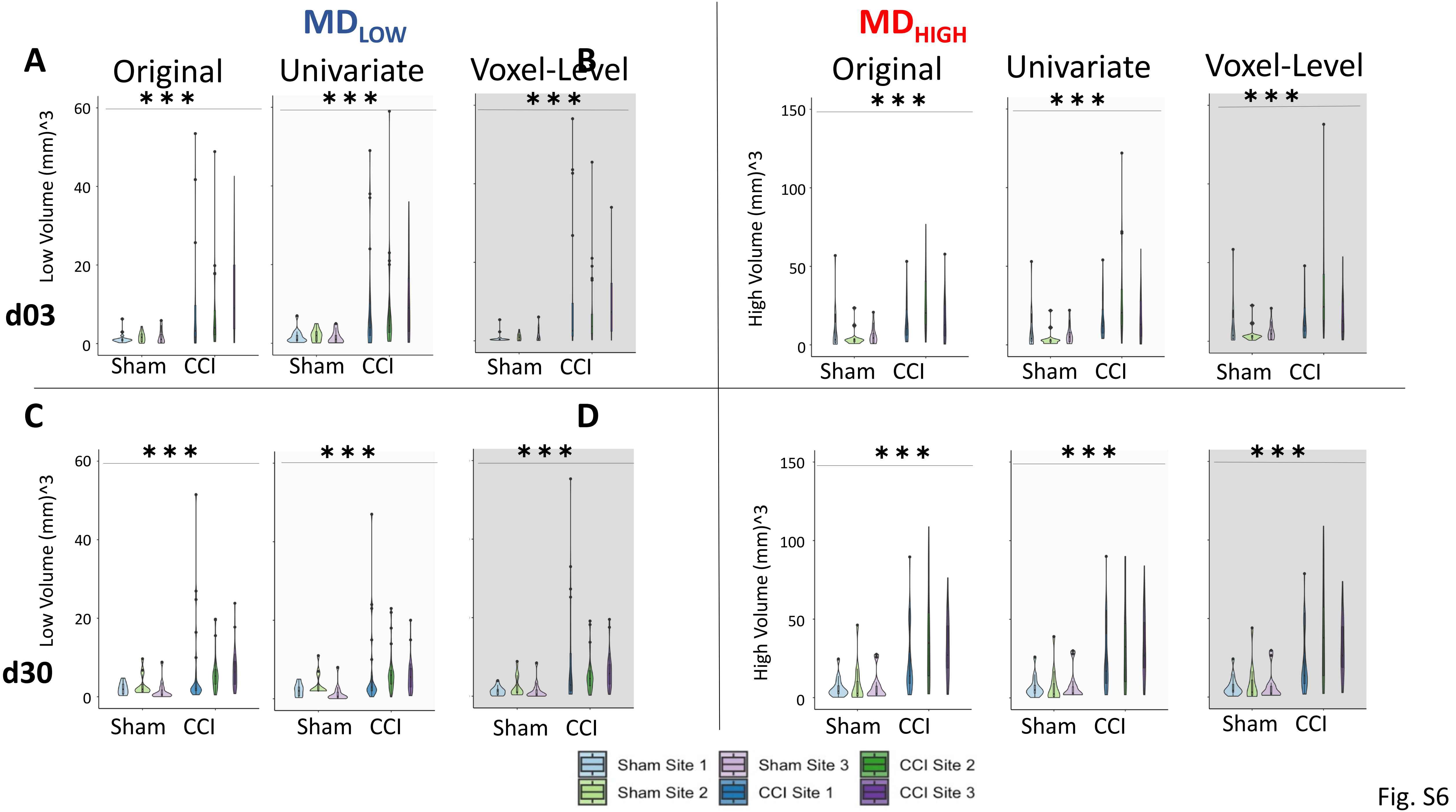
MDLOW
MDHIGH
A
B
C
D
Original
Univariate
Voxel-Level
d03
d30
***
Low Volume (mm)^3
High Volume (mm)^3
0
20
40
60
50
100
150
Sham
CCI
Sham Site 1
Sham Site 2
Sham Site 3
CCI Site 1
CCI Site 2
CCI Site 3

Fig. S6

Mean-Difference Plots: MD

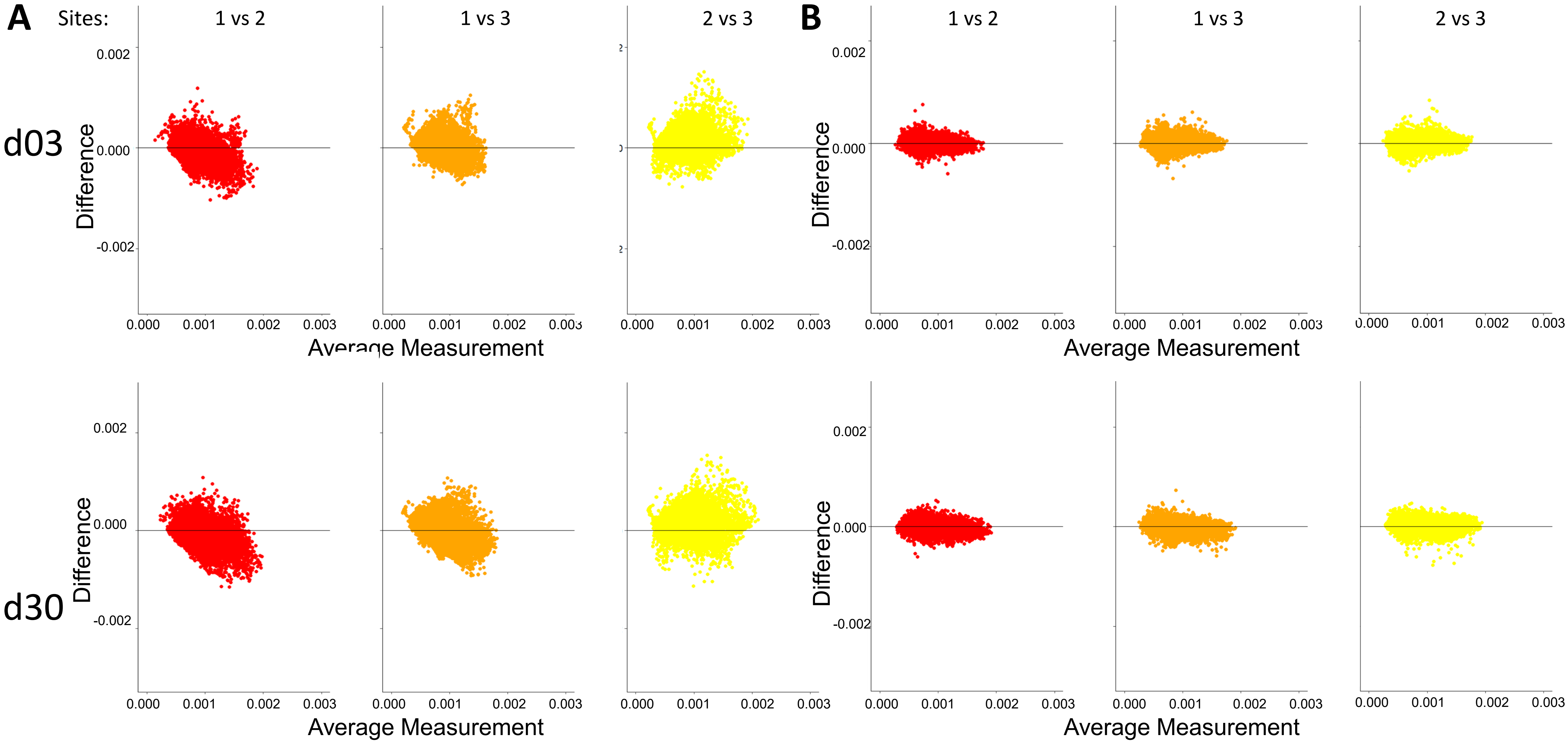

Fig. S7

Change in Power: MD

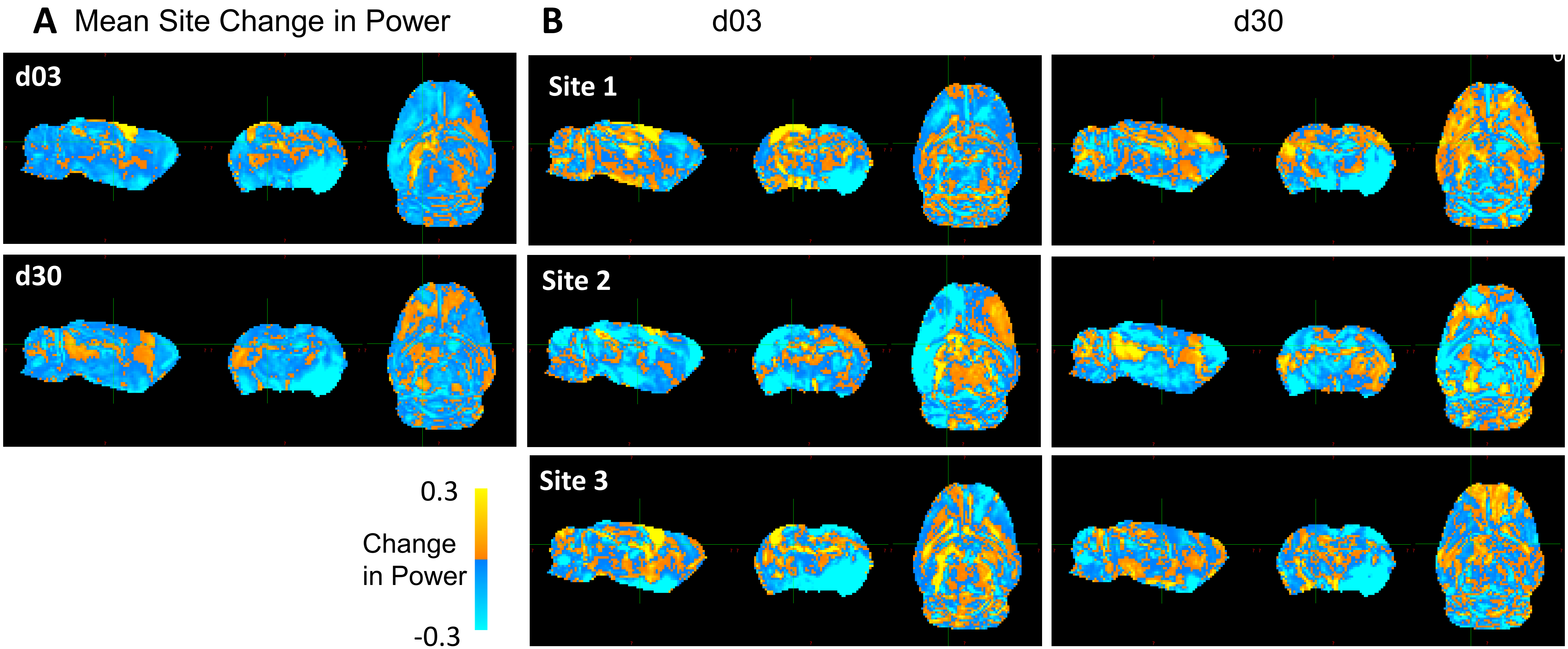

Fig. S8

Effect Size: MD

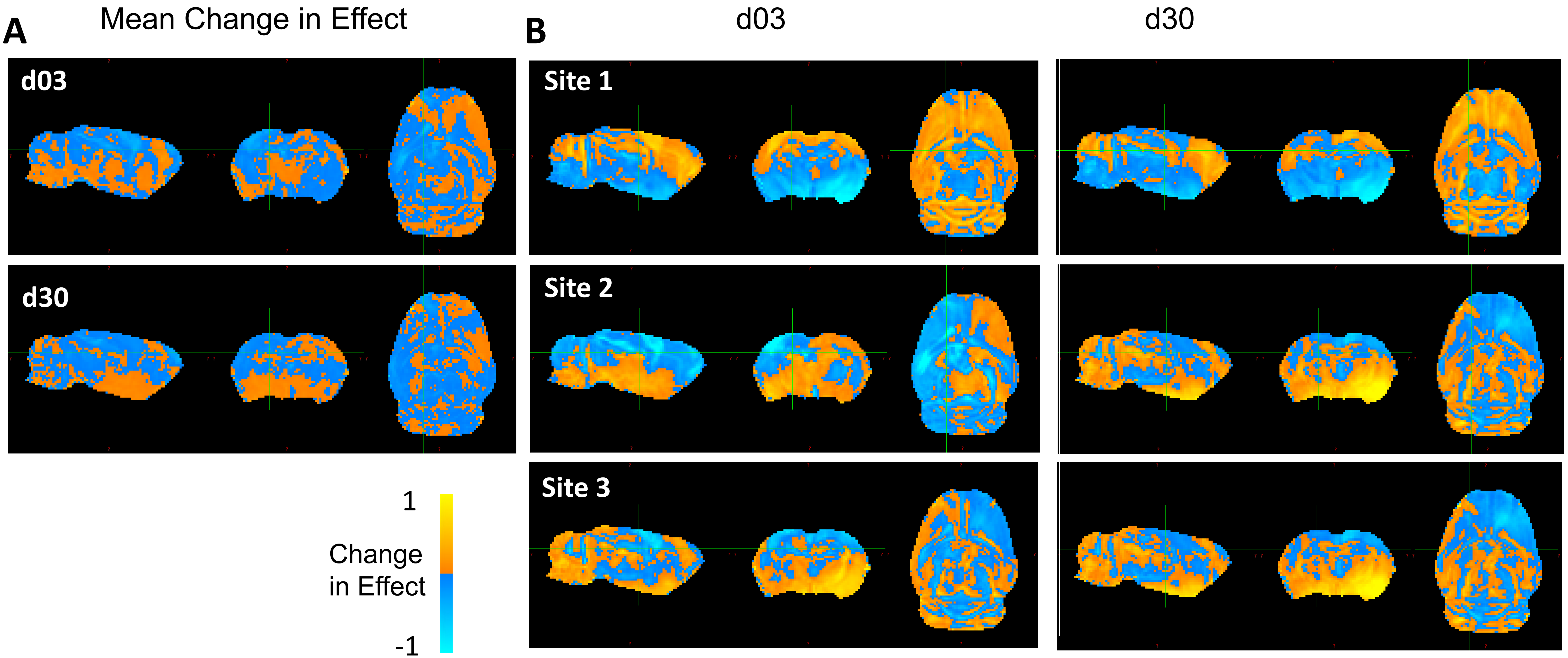

Fig. S9

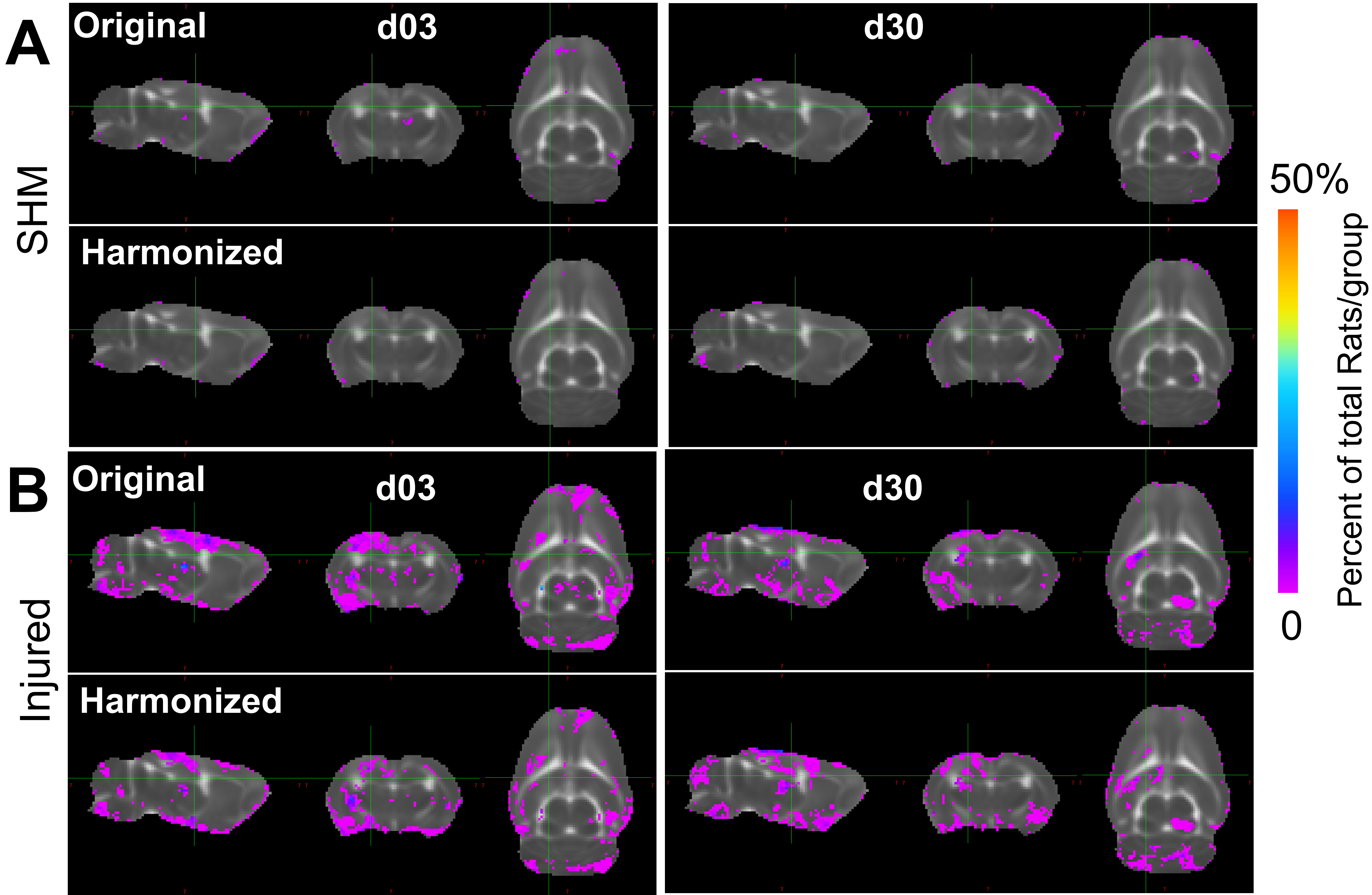
A
Original
d03
d30
SHM
Harmonized
50%
Percent of total Rats/group
0
B
Original
d03
d30
Injured
Harmonized

Fig. S10

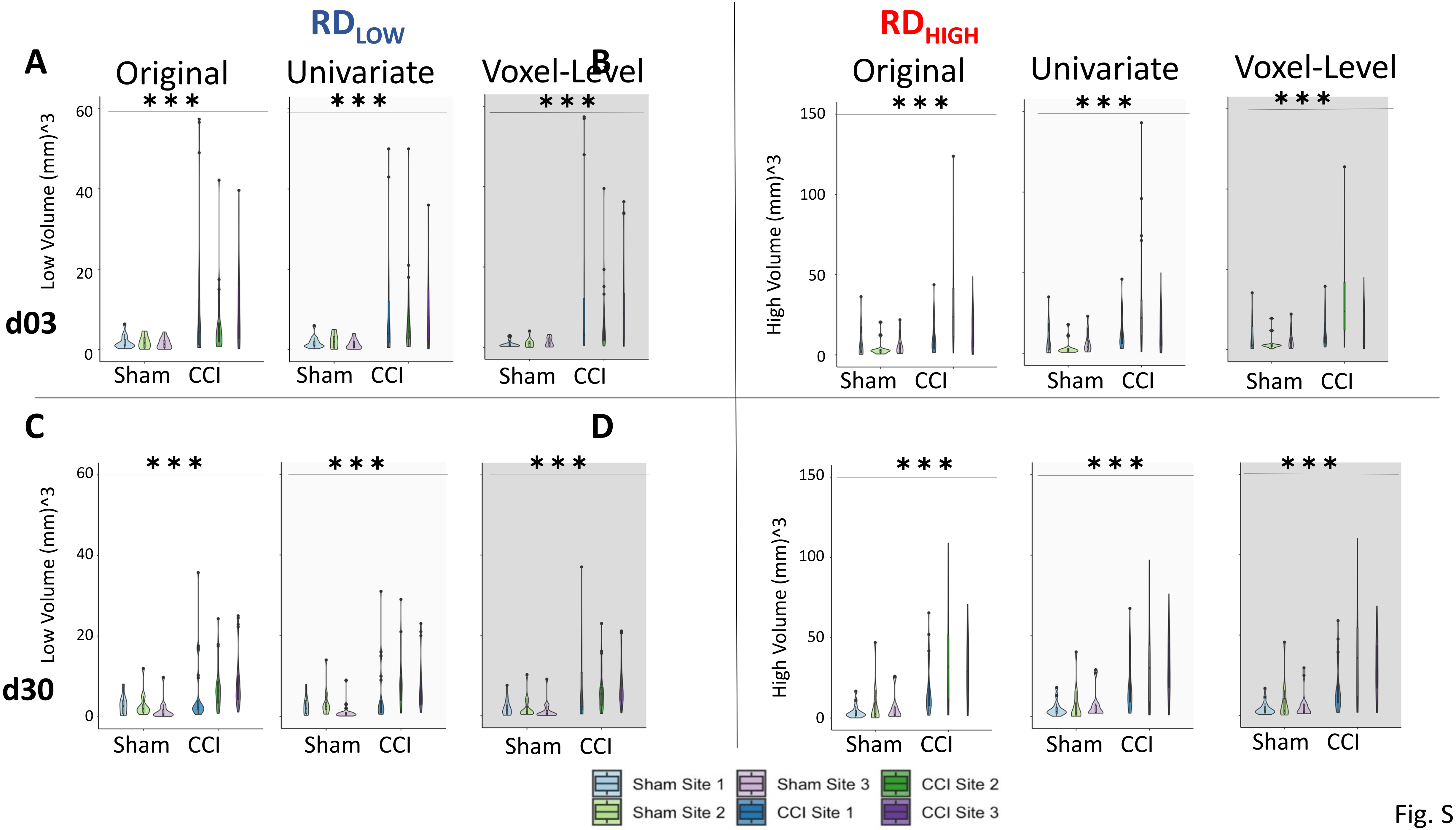

RDLOW
RDHIGH
A
B
C
D
Original
Univariate
Voxel-Level
* * *
Low Volume (mm)^3
High Volume (mm)^3
0
20
40
60
50
100
150
Sham
CCI
d03
d30
Sham Site 1
Sham Site 2
Sham Site 3
CCI Site 1
CCI Site 2
CCI Site 3

Fig. S11

Mean-Difference Plots: RD

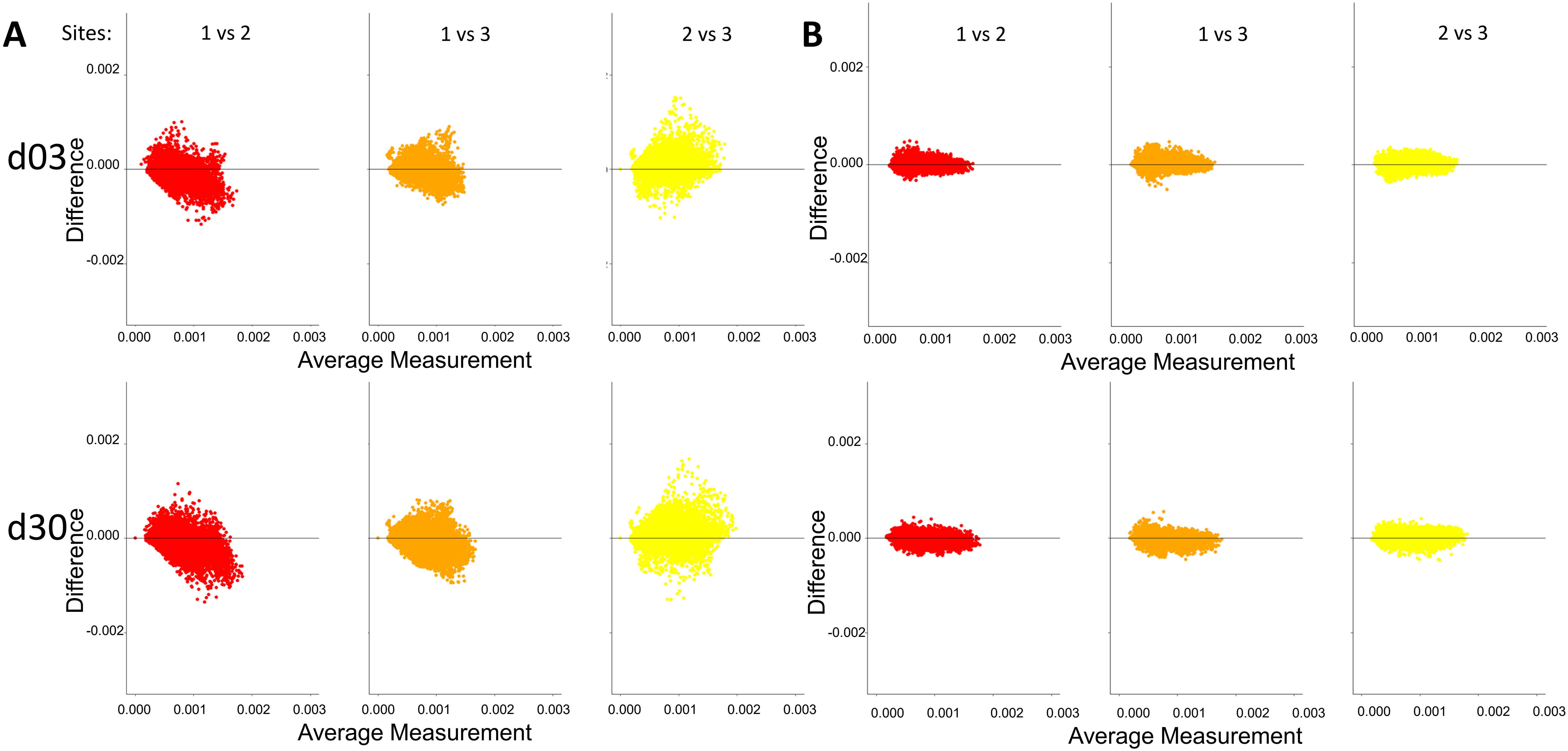

Fig. S12

Change in Power: RD

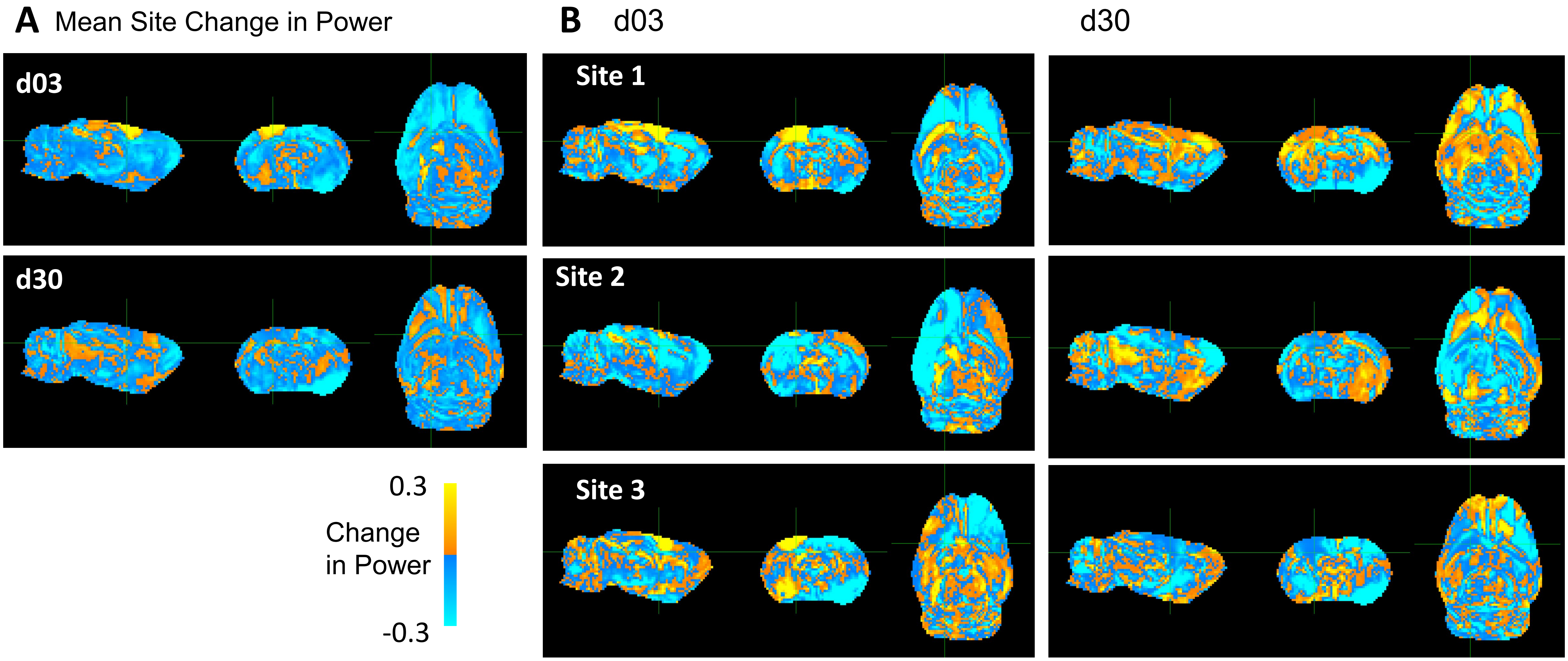

Fig. S13

Effect Size: RD

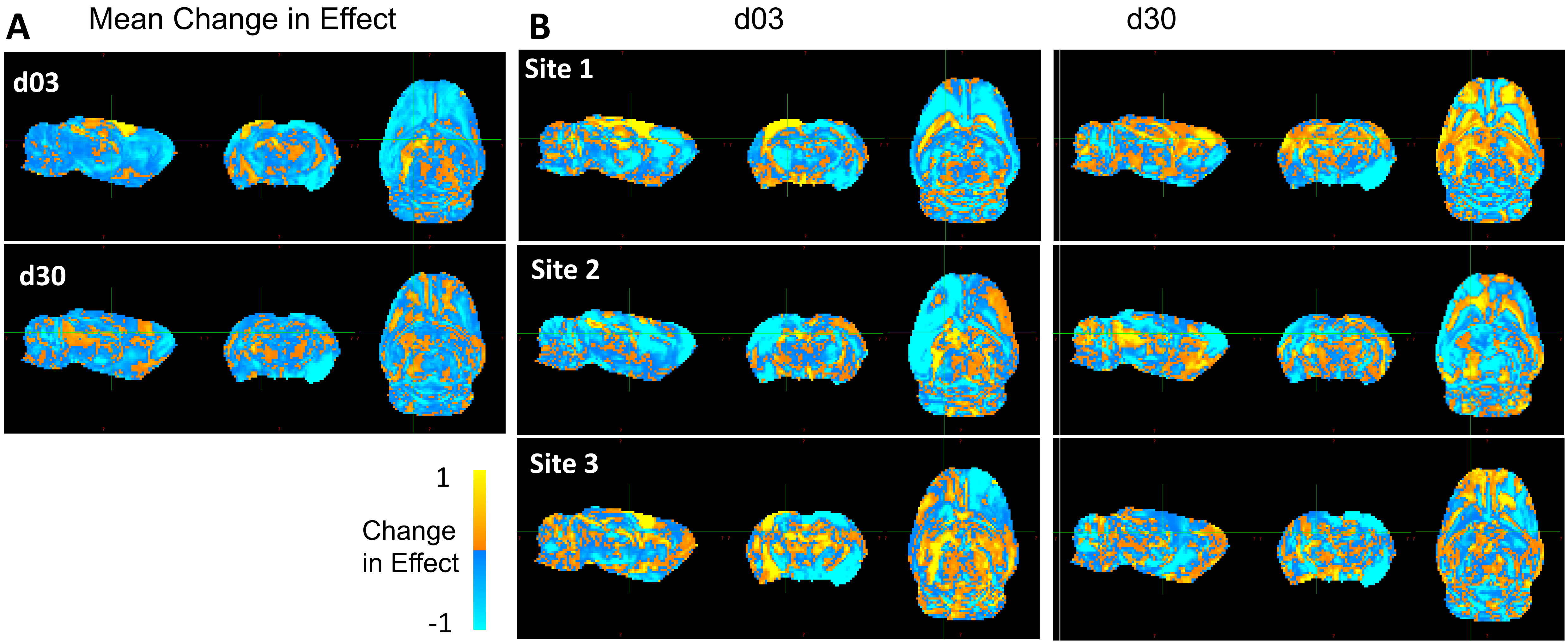

Fig. S14

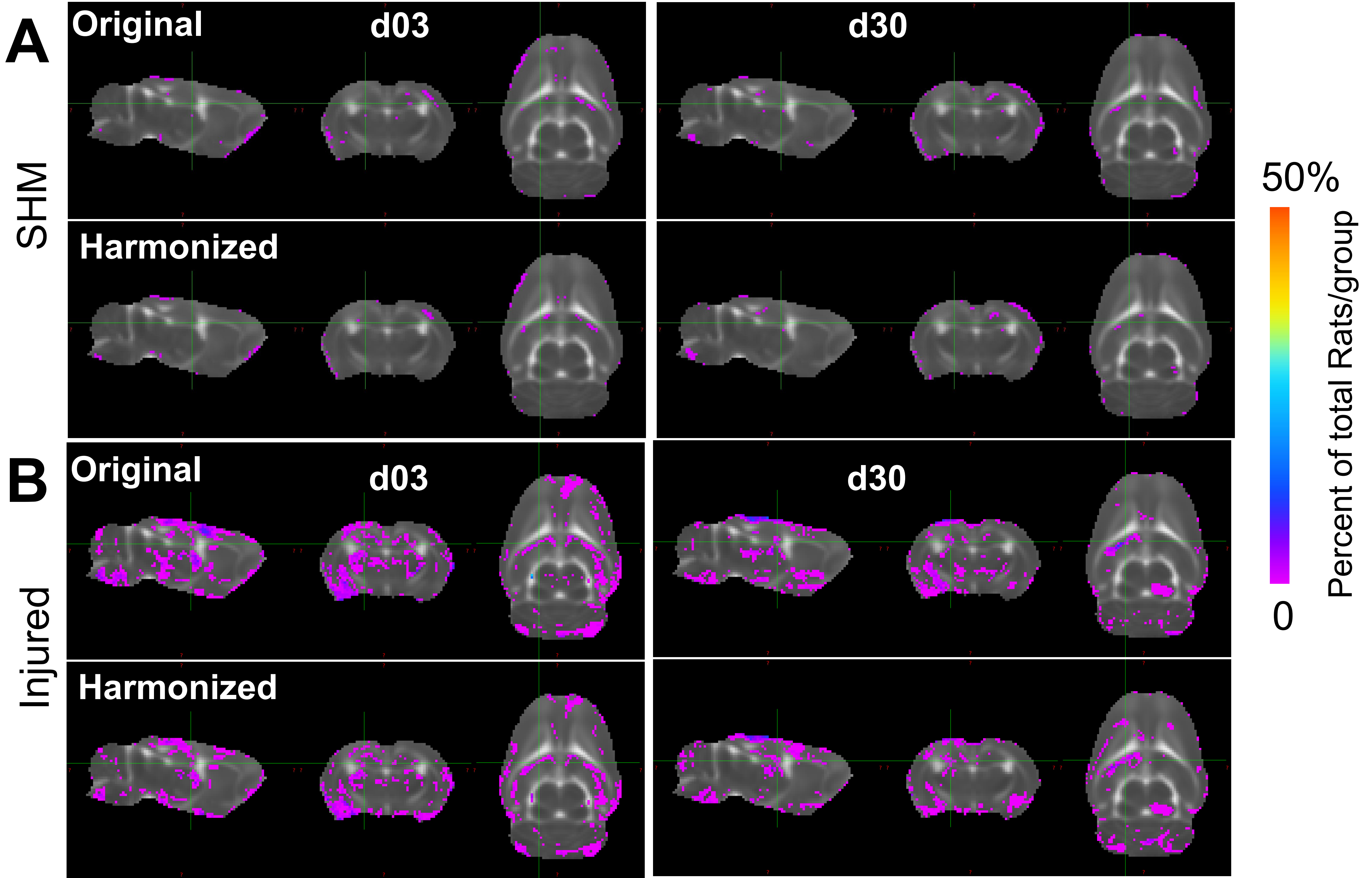
A
Original
d03
d30
SHM
Harmonized
B
Original
d03
d30
Injured
Harmonized
50%
0
Percent of total Rats/group

Fig. S15

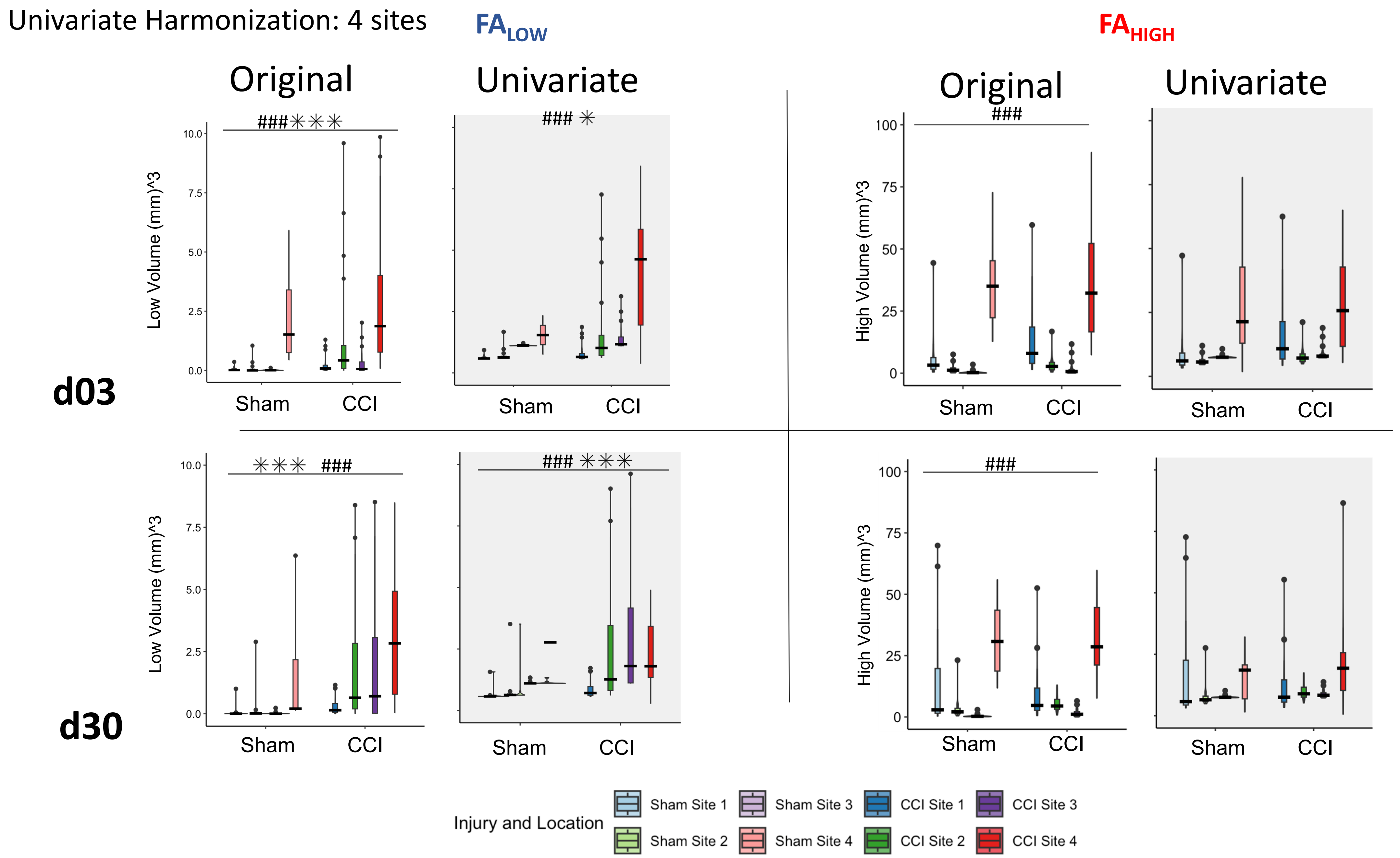

Univariate Harmonization: 4 sites
FA LOW
FA HIGH
Original
Univariate
Original
Univariate
d03
d30
###✳✳✳
✳
###
✳✳✳ ###
✳✳✳
###
Low Volume (mm)^3
High Volume (mm)^3
Sham
CCI
Injury and Location
Sham Site 1
Sham Site 2
Sham Site 3
Sham Site 4
CCI Site 1
CCI Site 2
CCI Site 3
CCI Site 4

Fig. S16

Change in Power: 4 sites

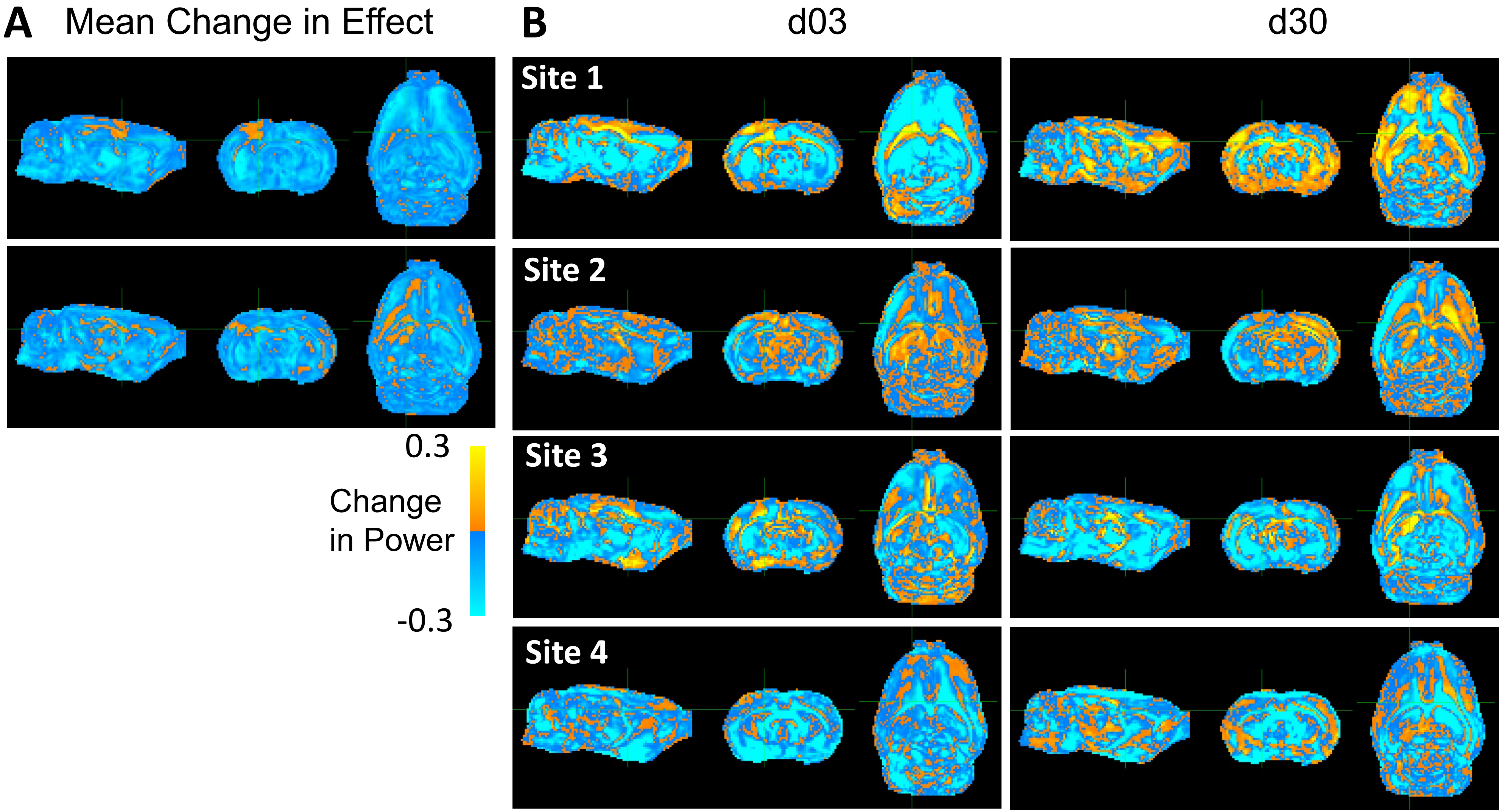

Fig. S17

Overlap Maps: 4 sites

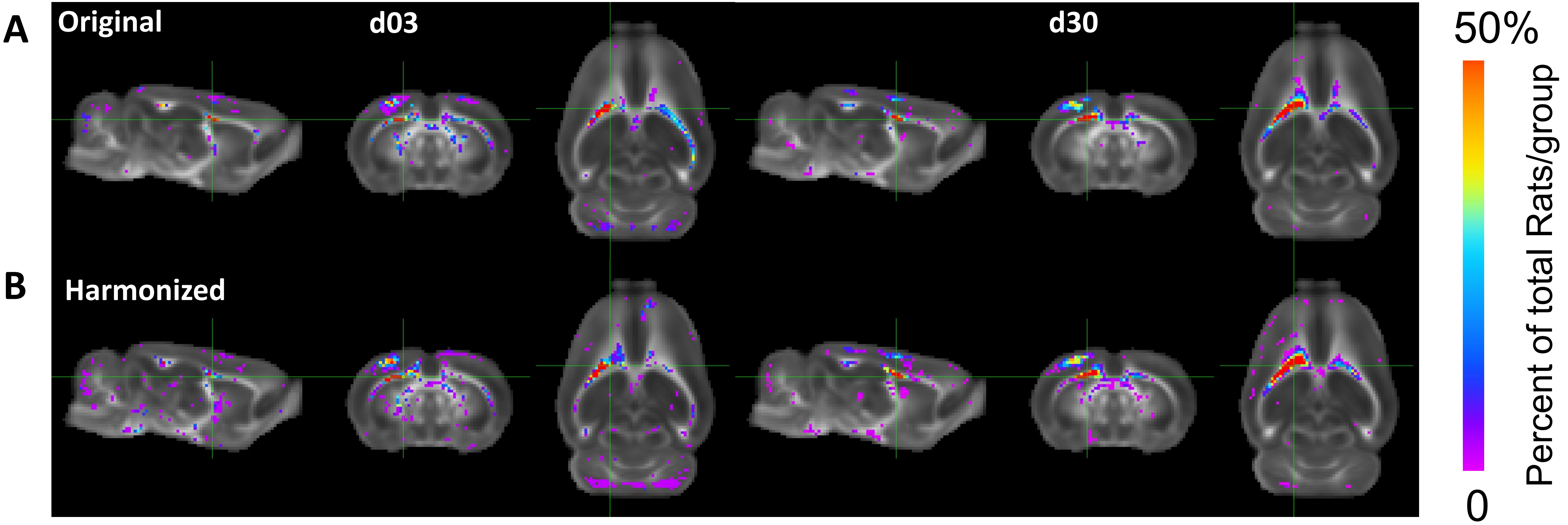

Fig. S18
